# Visualising disease trajectories from population-wide data

**DOI:** 10.3389/fbinf.2023.1112113

**Published:** 2023-02-08

**Authors:** Jessica Xin Hjaltelin, Hannah Currant, Isabella Friis Jørgensen, Søren Brunak

**Affiliations:** ^1^ Novo Nordisk Foundation Center for Protein Research, Faculty of Health and Medical Sciences, University of Copenhagen, Copenhagen, Denmark; ^2^ Copenhagen University Hospital, Rigshospitalet, Copenhagen, Denmark

**Keywords:** disease trajectory, visualisation, networks, personalised medicine, pancreatic cancer, comorbidity, multimorbidity, sankey

## Introduction

The rise of precision medicine as both a research discipline and clinical practice looks to improve clinical experience by using the wealth of health-associated data to tailor treatments towards the individual patient. Such data includes electronic health records alongside omics data that are increasingly collected in healthcare settings including genetic data, proteomics, metabolomics and more. Digitalisation of electronic healthcare has led to a wealth of data describing individuals’ health and disease status. Taken together, such data that describes individuals molecular biology and pathology is being used to expand our understanding and utility of personalised medicine.

Using electronic healthcare records and disease histories for either explorative or predictive purposes can help identify risk factors and stratify patients by disease risk which can ultimately inform screening protocols. In the recent decade, studies have been utilising the concept of disease trajectories in classical statistical approaches to explore risk factors and complications, deep learning algorithms for disease onset prediction or patient stratification, amongst others (Jensen et al., 2017; Shickel et al., 2018; Hu et al., 2019; Lademann et al., 2019; Nielsen et al., 2019; Thorsen-Meyer et al., 2020; Placido et al., 2022). Many studies have previously analysed diseases in an either mono- or bidirectional manner. Today, trajectory analyses and visualisations can utilise temporal information in expanding large health data sets allowing for consideration of comorbidities for different patients. Comorbidity and multimorbidity refer to the presence of more than one disease in a single patient and has been increasingly recognised as a crucial consideration when diagnosing and treating patients (Hu et al., 2016).

Visualisation can be a powerful tool for understanding all steps in an analysis using large data sets across a temporal axis. Denmark is one of the leading countries in collecting decades of longitudinal population-wide health data. Denmark has a wealth of health registries for which patients can be linked on an individual level through the unique Central Person Register (CPR) identifier. One of the largest and most comprehensive national registries is the Danish National Patient Registry (NPR), which covers around 8.2 million Danish patients over nearly 45 years. The visualisation of this type of large health dataset can be a highly complex matter. Here, we will use pancreatic cancer as an example to visualise temporal disease patterns in the NPR, giving examples of different types of plots and tools useful for the overviewing and analysing large longitudinal health data.

Pancreatic cancer is one of the most lethal cancer types with a 5-year survival rate at only 8% (American Cancer Society, 2020) and has been estimated to become the second leading cause of cancer in 203010. Due to the lack of clear symptoms, this cancer type is often diagnosed at a later stage resulting in poor outcomes. Hence, the need for detecting early symptoms and risk factors is crucial. We will highlight some of the key forms of data visualisation methods utilised in the analysis of disease trajectories, the ways in which these enrich our understanding of the data and the conclusions drawn.

## Visualising a population

**FIGURE 1:**
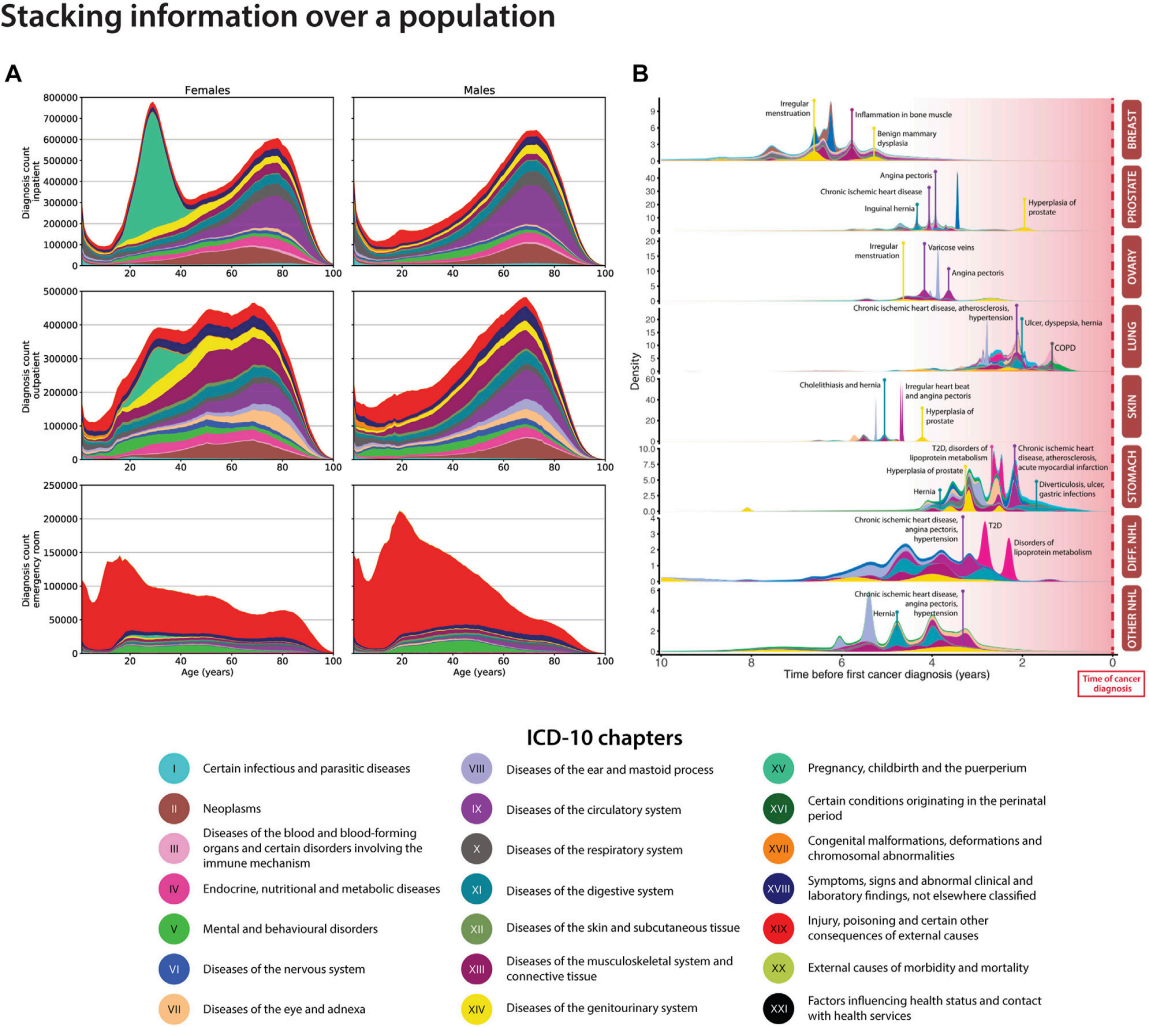
Density plots for visualising health data for the Danish population. **(A)**. The stacked density plot compares the amount of ICD-10 chapters between sexes for the entire Danish population *via* the National Patient Registry (NPR) (Adapted from Jensen et al. (2014). **(B).** The stacked density plot shows the occurrences of significant correlated disease pairs previous to top 8 cancer types (Hu et al., 2019). Breast: breast cancer; Prostate: prostate cancer; Ovary: ovarian cancer; Lung: lung cancer; Skin: skin cancer, Stomach: stomach cancer; Diff. NHL: diffuse non-Hodgkin’s lymphoma; Other non-Hodgkin’s lymphoma.

Visualisation is a powerful tool in understanding the cohort or population in which all analysis will take place, and to compare it to other populations. This visualisation can have the two-fold value: Checking that the data behaves as expected and is of high-quality; Identify novel patterns that may be indicative of unique biology or disease mechanism. For example, in the analysis of electronic healthcare data, an initial analysis may be to look at the distribution of different diagnosis types across age (Figure 1A). In Denmark, disease diagnoses are registered nation-wide in the NPR by the International Classification of Diseases version 8 and most recently (Rahib et al., 2014) (ICD-10). These are coded in electronic registries alongside the date of the diagnosis and patient birth information, from which the age of diagnosis can be derived. This can be plotted as a stacked density plot, and further stratified by sex and age (Figure 1A, ICD-10 period only) (Jensen et al., 2014). From Figure 1A we are able to notice general overview trends for the cohort such as the pregnancy chapter, emergency room contacts at younger ages and the increasing cardiovascular diagnoses from age 60. Stacked density plots can also be used to gain an overview of comorbidities along a temporal axis that represents a relative time since diagnosis of interest. For example, Figure 1B shows an overview of significant diagnoses (coloured by ICD-10 chapters) in the years up till a cancer diagnoses. This allows for the mapping of potential risk factors on a time scale, gaining a temporal trajectory. For example, we are able to see that as one might expect, in both breast cancer and ovarian cancer, irregular menstruation is observed in numerous patients prior to the diagnosis. Further, in cancers including that of the stomach and diffuse large B cell lymphoma (Diff. NHL), we observe a type 2 diabetes (T2D) diagnosis prior to the cancer diagnosis. This summarises not only disease pairs across a nation-wide cancer landscape, but also visualises them on a temporal scale prior to the event of interest.

## The disease trajectory highway and temporality

**FIGURE 2:**
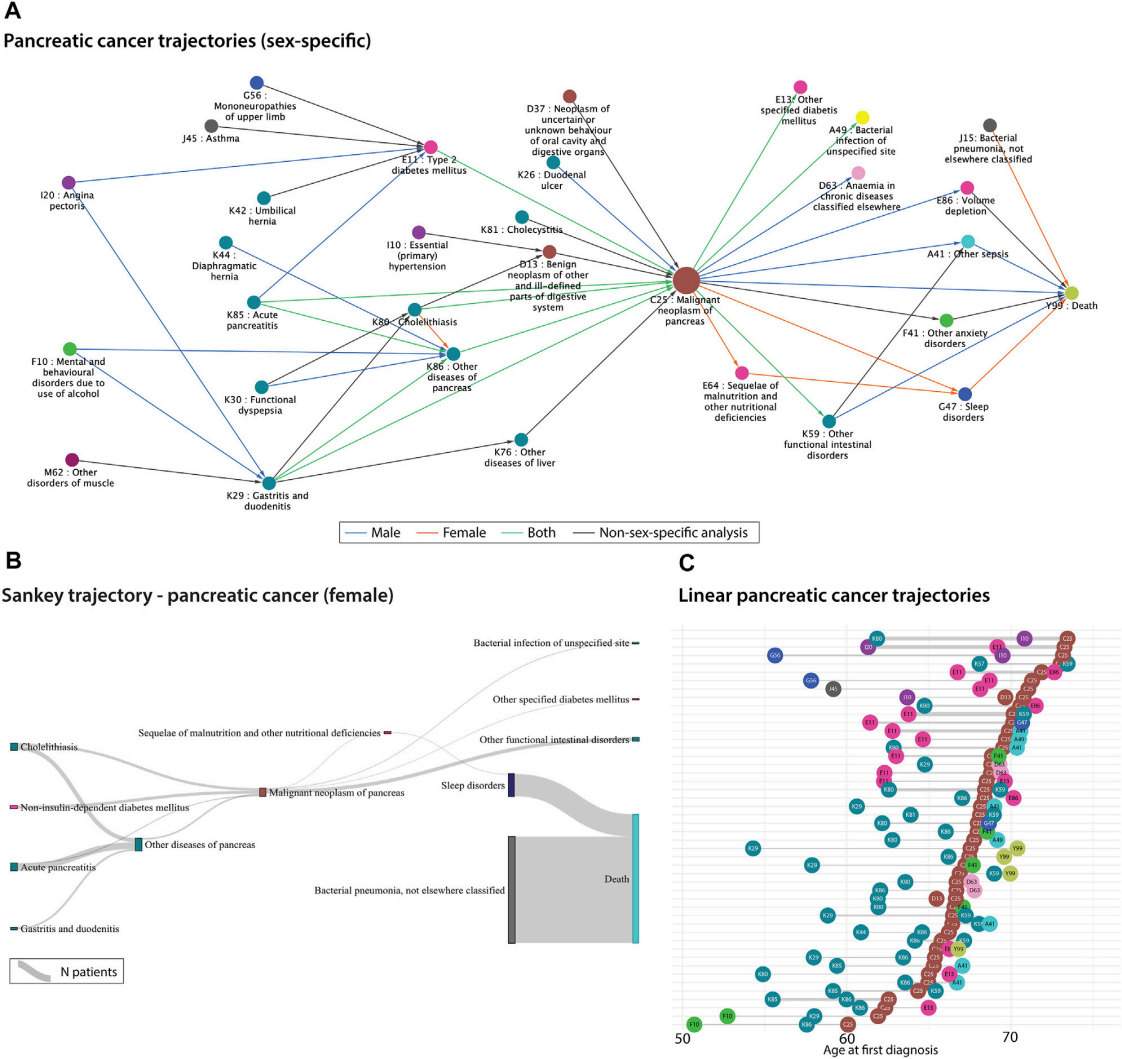
**(A)**. Sex-specific significant pancreatic cancer trajectories extracted from 7.2 million Danish disease histories from the National Danish Patient Registry (NPR). The correlations were extracted from the Danish Disease Trajectory Browser (DTB) (Siggaard et al., 2020). Edges are coloured according to sex-specific analyses run by the DTB. **(B)**. Sankey plot of the significant female-specific pancreatic cancer trajectories generated based on 7.2 million Danish disease histories from the National Danish Patient Registry (NPR). The disease-disease correlations were extracted from the publicly available Danish Disease Trajectory browser (Siggaard et al., 2020). The thickness of the links represents the number of patients. **(C)**. Linear trajectories plot showing 47 of the significant pancreatic cancer trajectories with three consecutive diseases. The temporal order of diagnoses is here represented as the mean age at the first diagnosis for patients that follow each of the trajectories. The thickness of the links represents the number of patients. The colours from all three figures represent the ICD-10 chapters (see Figure 1 legend).

Disease trajectories are longitudinal sequences of diseases that occur in a temporal order. Diseases could for example be represented by ICD-10 codes, symptom codes, text mined disease codes or symptoms (Jensen et al., 2017), (Jensen et al., 2014; Beck et al., 2016; Siggaard et al., 2020). The temporality of diseases can be very useful to stratify patients into different risk groups, understand comorbidities and multimorbidities or improve disease progression patterns. Examples of how to visualise diseases using a network view could be *via* the Cytoscape software (Shannon et al., 2003) or the Danish Disease Trajectory Browser (for Danish disease correlations) (Siggaard et al., 2020). For the latter, population-wide summarised data from the NPR can be collected to visualise significant disease trajectories for a disease of interest. Figure 2A shows a network extracted from the Danish Disease Trajectory Browser for pancreatic cancer patients. The network nodes are coloured in chapters according to the ICD-10 chapters and edges are coloured according to sex-specific disease-disease correlations. For this example, we can see that male-specific correlations involve angina pectoris and alcohol abuse disorders, while female patients have post-cancer malnutrition deficiencies and sleep disorders. Although the directional correlations are significant in the analysis (Siggaard et al., 2020), they have not been proven to be causal.

Another useful visualisation method for patient or disease trajectories are Sankey and alluvial flow diagrams. The width of the diagram bars conveys the number of patients in a specific link. We used publicly available pancreatic cancer-specific disease correlations from the Danish Disease Trajectory Browser (for Danish disease correlations) (Siggaard et al., 2020) to visualise patient groups flowing across disease states. One should be aware that alluvial and Sankey diagrams have different underlying assumptions. One difference is for example that alluvial diagrams have aligned bars in columns/dimensions, whereas the bars in Sankey plots can be distributed anywhere, depending on the specific Sankey algorithm. In Figure 2B, trajectories are combined by disease pairs using significant pancreatic cancer disease pairs from the DTB. Here, the disease pairs have been linked by the Sankey algorithm, thus it may not be the same patient group that traverses an entire trajectory. These types of flow diagrams are getting more focus for visualising longitudinal healthcare data such as prescription trajectories (Aguayo-Orozco et al., 2021), symptom trajectories (Lademann et al., 2019), cancer trajectories (Hu et al., 2019), hospital flow from acute coronary syndrome (Pinaire et al., 2021) etc.

Disease trajectory networks can be useful to get an overview of the alternative disease routes for patients. Even though, the trajectories are temporal, Figures 2A,B do not inform about the time between the diagnoses. Figure 2C visualises single linear disease trajectories as a function of time. It is represented as the mean age at the first diagnosis for the patients following the specific trajectory and thereafter, sorted using the mean age of pancreatic cancer. Thus, one can investigate the time between diagnoses and the average age of diagnosis. The pancreatic cancer diagnosis often appears after the age of 60 which is consistent with a late diagnosis. For example, diseases from “diseases of the digestive system” chapter (cyan coloured nodes) seem to appear earlier than type II diabetes (pink node E11) in relation to pancreatic cancer, which could be valuable to consider when developing screening protocols or tools. The poor prognosis of pancreatic cancer is also visualised here, since some trajectories include death shortly after the pancreatic cancer diagnosis. Disease trajectories can be combined with mortality information to stratify patients and optimally improve treatment or surveillance for these patient groups (Beck et al., 2016; Shang et al., 2022; Yang et al., 2019).

## Final considerations

With the constantly increasing amounts of data within healthcare and research, there is a huge need for improved and more dynamic and interactive visualisation tools. Most visuals today are static images. But the complexity of data that expands by both velocity, variety and volume, needs new methods for comprehending, analysing and interpreting them in the multidimensional spaces they live in.

Increasingly, studies are using disease trajectories together with deep learning models for risk prediction and stratification of patients. Here, a big challenge is to visualise and explain temporal patterns picked up by these models, which is essential for applying them to decision-making in the clinics. Currently, some tools have been developed to target this problem including SHAPley values, deepExplain and others (Lundberg and Lee, 2017). Although the “static” networks shown above visualises data from a certain time interval only, another task will be to develop models and visualisation of patient’s disease progression in real time. This is particularly important within intensive care, where the data richness is much higher than in the diagnosis trajectories shown here. Due to the emergence of wearable data all patients will with time grow in data richness begging the development of live models of high complexity.
